# Cloning and *in vitro* characterization of a *Schistosoma japonicum* aquaglyceroporin that functions in osmoregulation

**DOI:** 10.1038/srep35030

**Published:** 2016-10-13

**Authors:** Yuzheng Huang, Wei Li, Wuguang Lu, Chunrong Xiong, Yang Yang, Huaijiang Yan, Kun Connie Liu, Peng Cao

**Affiliations:** 1 Affiliated Hospital of Integrated Traditional Chinese and Western Medicine, Nanjing University of Chinese Medicine, Nanjing, Jiangsu 210028, China; 2 Jiangsu Province Academy of Traditional Chinese Medicine, Nanjing, Jiangsu 210028, China; 3 Jiangsu Institute of Parasitic Diseases, key Laboratory on Technology for Parasitic Diseases Prevention and Control, Ministry of Health, Wuxi, Jiangsu 214064, China; 4 Johns Hopkins Malaria Research Institute, Dept. Molecular Microbiology and Immunology, Bloomberg School of Public Health, The Johns Hopkins University, Baltimore, MD 21205, USA

## Abstract

As one of the three major human pathogens that cause schistosomiasis, *Schistosoma japonicum* is the only one that is endemic in China. Despite great progress on schistosomiasis control over the past 50 years in China, *S. japonicum* transmission still occurs in certain endemic regions, which causes significant public health problems and enormous economic losses. During different life stages, parasites are able to survive dramatic osmolality changes between its vector, fresh water, and mammal host. However, the molecular mechanism of parasite osmoregulation remains unknown. To address this challenging question, we report the first cloning of an *S. japonicum* aquaglyceroporin (*Sj*AQP) from an isolate from Jiangsu province, China. Expressing *Sj*AQP in *Xenopus* oocytes facilitated the permeation of water, glycerol, and urea. The water permeability of *Sj*AQP was inhibited by 1 mM HgCl_2_, 3 mM tetraethylammonium, 1 mM ZnCl_2_, and 1 mM CuSO_4_. *Sj*AQP was constitutively expressed throughout the *S. japonicum* life cycle, including in the egg, miracidia, cercaria, and adult stages. The highest expression was detected during the infective cercaria stage. Our results suggest that *Sj*AQP plays a role in osmoregulation throughout the *S. japonicum* life cycle, especially during cercariae transformation, which enables parasites to survive osmotic challenges.

Schistosomiasis is a neglected tropical disease that is caused by Platyhelminthes of the *Schistosoma* genus. It remains one of the most serious parasitic diseases in clinics and public health, especially in Asia, south America and Africa^1^,^2^. In 2014, at least 258 million people required preventive treatment and 61.6 million people were treated for schistosomiasis^3^. As one of the three major causative agents of human schistosomiasis, *Schistosoma japonicum* is the most malignant and the only human blood fluke that is endemic in regions of China, the Philippines, and parts of Indonesia^4^,^5^. It has more than 40 kinds of potential hosts that serve as reservoirs for human infections, and this unique feature complicates the transmission patterns of *S. japonicum*. *S. japonicum* infection leads to Katayama fever, as well as liver fibrosis, cirrhosis, portal hypertension, and splenomegaly. Repeated infections also cause chronic impairment of the liver^3^,^6^. Despite the remarkable success of schistosomiasis control over the past 50 years in China, this disease still remains endemic in certain lake and marshland regions, and it causes significant public health problems and enormous economic losses^7^,^8^; Hu *et al*. 2015). Compared with intensive epidemiological surveys, functional characterizations of proteins are important for understanding parasite physiology and transmission, especially the channel-forming aquaporins (AQPs), which have not been studied extensively and whose functions need to be determined to improve our knowledge of this important pathogen.

AQPs are a family of channel proteins that facilitate the movement of water and small neutral solutes across cell membranes. A deficiency of human AQPs causes disequilibria of water and solutes in the body, leading to clinical complications such as nephrogenic diabetes insipidus^9^,^10^. Widely distributed in nature, AQPs have been found in almost every known organism^9^,^11^. Based on their permeation specificities, aquaporins are further divided into two sub-families, water-selective AQPs or aquaglyceroporins. The former subgroup is permeable to water, while the latter exhibit permeability to small neutral solutes such as glycerol and urea, as well as water^9^,^12^. Aquaglyceroporins are the only known glycerol channels in mammals, and they play essential roles in osmoregulation by facilitating the import or export of glycerol, a major intracellular osmolyte^13^. The expression of certain aquaglyceroporins is up-regulated up to 20-fold under stresses such as low osmolality^13^, heat^14^, cold^15^, or starvation^16^. Glycerol is the major osmolyte, and its content in yeast cells increases under restrict regulation of the mitogen-activated protein kinase pathway, which increases intracellular osmolality and helps yeast survive osmotic stresses^17^. Moreover, glycerol is also a well-known stabilizer that protects proteins against denaturation through preferential hydration^18^,^19^.

*S. japonicum* has a complex life cycle that requires transformations among the free-living stage in fresh water, and intracellular stages in intermediate vectors or hosts^20^. It must have developed a mechanism to adapt to different environments, which may provide a novel and specific means of schistosomiasis control. However, thus far, there have been no reports of *S. japonicum* osmoregulation at the molecular level. The objectives of the present study were to clone and characterize the functions of an AQP in *S. japonicum* (hereafter *Sj*AQP), as well as to determine its expression pattern throughout the parasite life cycle. Our results reveal the contributions of *Sj*AQP to parasite survival during dramatic osmolality changes.

## Materials and Methods

### Ethics statement

All experiments using the *S. japonicum* parasite, *Oncomelania hupensis* snails, and mice were performed under protocols approved by the Jiangsu Parasitic Disease Institute (Wuxi, Jiangsu Province, China) with China guidelines (permit no. [2006]398). Parasite and snail was approved by the Biological Studies Animal Care and Use Committee, Peoples Republic of China. All the methods were performed in accordance with the relevant guidelines and regulations of China.

### Parasites, animals, protocols, sequences, and phylogenesis

Recombinant DNA procedures were performed by protocols approved by the Johns Hopkins University with National Institutes of Health (NIH) guidance. Cercariae were removed from *O. hupensis* snails that were artificially infected with *S. japonicum*. Each BALB/c mouse was percutaneously infected with 30 *S. japonicum* cercariae through shaved abdominal skin. Adult *S. japonicum* worms were later harvested by portal perfusion of infected mice at 35 d post-infection. Eggs were collected from dissected livers of infected mice, and then hatched into miracidia. *O. hupensis* snails were cultivated under standard protocols in the Jiangsu Parasitic Disease Institute, exposed to *S. japonicum* miracidia for infection, and then harvested for RNA isolation and other molecular biology experiments.

The mRNA sequence of *Sj*AQP determined in this study was submitted to the National Center for Biotechnology Information (NCBI) (GenBank accession no. KR709301.1). Detailed methods of phylogenetic analysis, RNA isolation, reverse transcription (RT), as well as quantitative polymerase chain reaction (qPCR) with the delta-delta Ct analysis were described in a previous paper^21^. Briefly, SYBR Green qPCR master mix (Applied Biosystems, Foster City, CA, USA) was used. Each 25-μl reaction was repeated in triplicate. The optimized qPCR program was one cycle at 50 °C for 2 min, followed by 1 cycle of 95 °C for 2 min, followed by 40 cycles of 95 °C for 30 s, 60 °C for 30 s, and 72 °C for 30 s, followed by a final 10-min elongation step at 72 °C. Primer concentrations were 200 nM. Specific primers designed for *Sj*AQP cloning, qPCR, and quality controls are listed in Table 1. The NADH gene is one of the most stably expressed housekeeping genes under different developmental stages^22^; thus, it was chosen as the control for qPCR normalizations.

The sequence of the primers for cloning AQP in our research was designed based on the published mRNA sequence (GenBank: AY813118.1)^23^. AQP sequences and GenBank accession numbers used in our phylogenetic analysis are: from *Homo sapiens* and *Bos taurus* hosts (*Hs*AQP1, NP_001171989.1; *Bt*AQP1, NP_001073262.1); the liver fluke *Opisthorchis viverrini* (*Ov*AQP1, KF697690; OvAQP2 KF697691); *Schistosoma mansoni* (*Sm*AQP ACI31185.1); the Chinese liver fluke *Clonorchis sinensis* (*Cs*AQP3, GAA31414; *Cs*AQP9, GAA55320); the rodent malaria-causing agent *Plasmodium berghei* (*Pb*AQP #XM_671432); the malignant human malaria pathogen *Plasmodium falciparum* (*Pf*AQP #AJ413249); and *Trypanosome brucei* (TbAQP1, 2, and 3, AJ697889, AJ697890, and AJ697891, respectively). A multiple sequence alignment was performed with ClustalW. The phylogenetic tree is presented using pairwise scores, which are simply the number of identities between two sequences, divided by the length of the alignment, and they are represented as percentages. A neighbor-joining tree was created by ClustalW, downloaded, and presented by TreeView 0.5.0 software. According to the software provider, the unit of the phylogenetic tree represents 0.1 amino acid substitutions.

### *In vitro* complimentary RNA (cRNA) transcription, Xenopus oocyte injection, and osmotic swelling assays

Plasmid was constructed by ligating the *Sj*AQP fragment between the Bgl*II* sites of the pXβG-ev1 vector. cRNA transcription, oocyte preparation, microinjection, an osmotic swelling assay for water permeability measurement, and inhibition assays were described previously^24^. Glycerol and urea permeabilities were measured with previously described methods^16^. Briefly, cRNA of *Sj*AQP, without or with a carboxyl-terminal myc-tag, was *in vitro* transcribed using the linearized pXβG-*Sj*AQP plasmid as the template. The size and quality of the cRNA product were confirmed by denaturing gel electrophoresis. For protein expression in *Xenopus laevis* oocytes, 5 ng (in 69 nL) of cRNA were injected into each oocyte. Control oocytes were injected with the same volume of nuclease-free water. After growing in modified Barth’s solution for 3 d, oocytes were tested in osmotic swelling assays, and the coefficient of osmotic water permeability (P_f_) and solute permeability (P_s_) were determined as previously described^16^,^24^. Briefly, the relative volume (V/V_0_) was calculated from the area at the initial time (A_0_) and after a time interval (At) as follows: V/V_0_ = (At/A_0_)^3/2^. P_f_ was determined from the initial slope of the time course [d(V/V_0_)/dt], the initial oocyte volume (V_0_ = 9 × 10^−4^ cm^3^), the initial oocyte surface area (S = 0.045 cm^2^), and the molar volume of water (Vw = 18 cm^3^/mol) as follows: P_f_ = [Osmtotal × Vo × d(V/Vo)/dt]/[S × Vw × (Osm_in − Osm_out)]. Non-isotopic solute permeabilities (P_s_) were measured by placing oocytes in 200 mOsm modified Barth’s solution containing 100 mOsm of solute, which caused water influx and oocyte swelling. P_s_ was calculated from the oocyte surface area (S = 0.045 cm^2^), the initial oocyte volume (Vo = 9 × 10^−4^ cm^3^), the initial slope of the relative volume increase d(V/Vo)/dt, the total osmolality of the system (Osmtotal = 200 mOsm), and the osmotic solute gradient (Osm_out − Osm_in) as follows: P_s_ = [Osmtotal × Vo × d(V/Vo)/dt]/[S × (Osm_out − Osm_in)]. At least six individual oocytes were measured in each treatment, and statistical significance was determined by a Student’s *t*-test.

In the inhibition assays, oocytes were pre-incubated with inhibitors for 5 min at room temperature at the indicated final concentrations, and Pf and Ps were determined. The proper concentration of inhibitor ions tested in this study was determined by previous research on other AQPs by other groups^21^,^25^,^26^,^27^,^28^.

## Results

### *Sj*AQP cDNA and its deduced protein sequence

**Figure 1:**
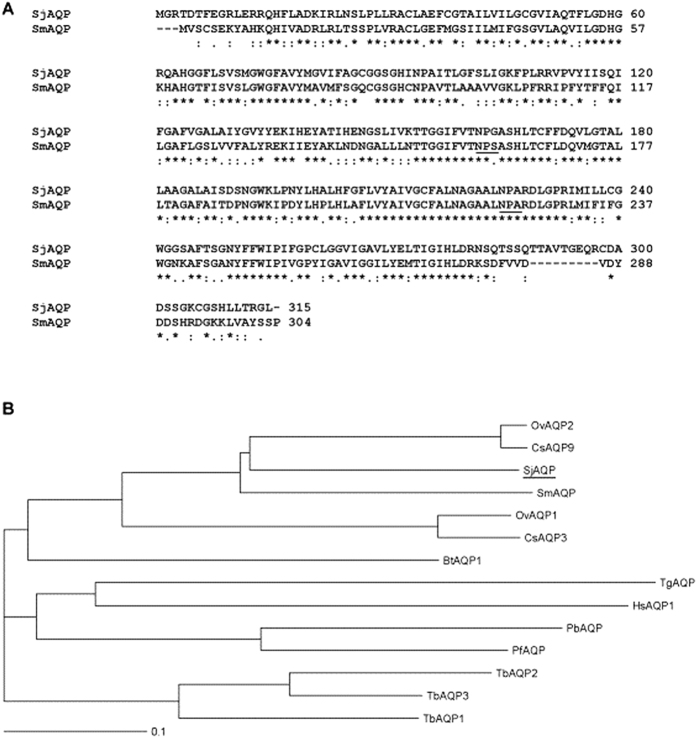
Deduced primary protein sequence and phylogenetic analysis of *Sj*AQP. (**A**) Deduced amino acid sequence of *Sj*AQP and alignment with its homolog *Sm*AQP from *Schistosoma mansoni*. Asterisks indicate fully conserved residues; colons indicate strongly conserved similar residues with scores >0.5 in the Gonnet PAM 250 matrix; and periods indicate weakly similar residues with scores ≤0.5 in the matrix. The two highly conserved loops of the AQP family are underlined. (**B**) Phylogenetic analysis of *Sj*AQP and characterized homologs from *Homo sapiens* (*Hs*AQP1), *Bos taurus* (*Bt*AQP1), *Plasmodium berghei* (*Pb*AQP), *Plasmodium falciparum* (*Pf*AQP), *Opisthorchis viverrini* (*Ov*AQP), *Clonorchis sinensis* (*Cs*AQP), *Toxoplasma gondii* (*Tg*AQP), and *Trypanosome brucei* (*Tb*AQP). Unit, 0.1 amino acid substitutions per site.

As shown in Fig. 1A, we determined the full-length *Sj*AQP cDNA sequence of a Jiangsu *S. japonicum* isolate. This mRNA sequence has been submitted to the NCBI under GenBank accession no. KR709301.1. The deduced protein sequence shares 59.5% identity with an *Sm*AQP (Fig. 1A). Typical AQPs contain two canonical Asn-Pro-Ala (NPA) motifs that line the pore region to restrict proton conductance. Interestingly, in *Sj*AQP, the first motif is Asn-Pro-Gly (NPG) rather than NPA. Similarly, the aquaglyceroporin *Sm*AQP also carries an amino acid exchange in the first loop, which replaces the conventional NPA motif with Asn-Pro-Ser (Fig. 1A, underlined). *Sj*AQP shares the highest sequence homology with homologs from *O*. *viverrini* (*Ov*AQP2), *C. sinensis* (*Cs*AQP9), and *S. mansoni* (*Sm*AQP). The amino acid sequence identities are 62.4, 62.1, and 59.5%, respectively, as shown in the phylogenetic tree in Fig. 1B. *Sj*AQP also shares 20.7 and 38.0% amino acid sequence identities with AQPs from humans (*Hs*AQP1) and bovines (*Bt*AQP1), respectively.

### Functional characterization of *Sj*AQP in Xenopus oocytes

**Figure 2:**
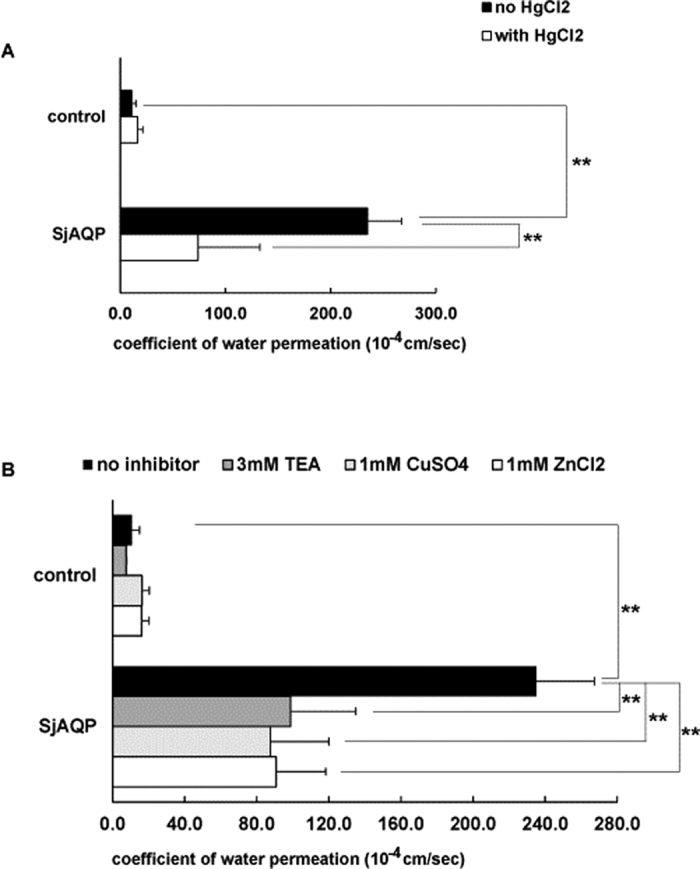
Water-permeating activity of *Sj*AQP. (**A**) *Sj*AQP-expressing oocytes significantly facilitate water movement across cell membranes, and the permeation is significantly inhibited by 1.0 mM HgCl_2_. Black and white bars represent permeation coefficients of control or *Sj*AQP-expressing oocytes, respectively. (**B**) Water permeation by *Sj*AQP-expressing oocytes is significantly inhibited by 3.0 mM tetraethylammonium, 1.0 mM CuSO_4_, and 1.0 mM ZnCl_2_. The *x*-axis is the coefficient of osmotic water permeability, P_f_, with the unit 10^−4^ cm/s. Data are represented as means ± SDs. *p ≤ 0.05, **p ≤ 0.01 by a Student’s *t*-test.

**Figure 3:**
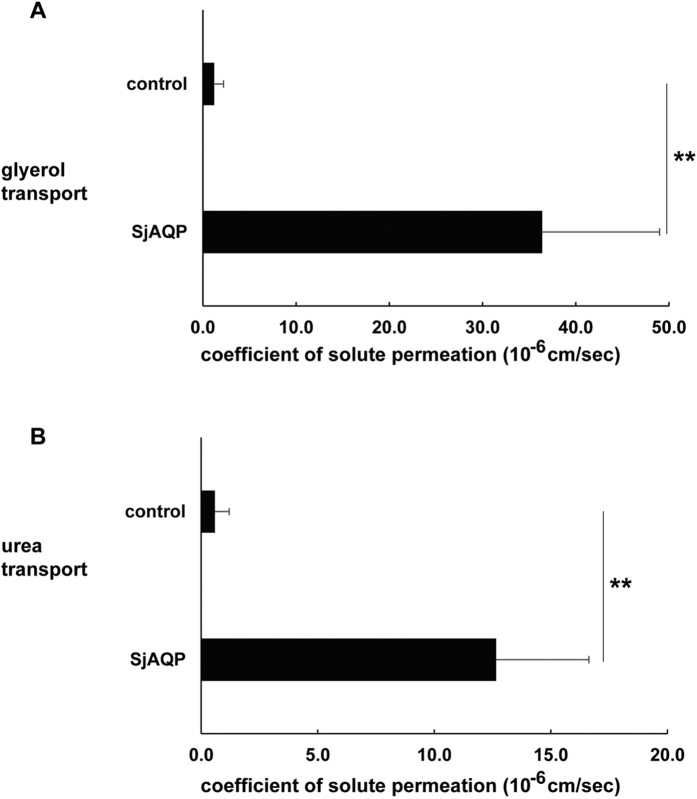
Solute-permeating activities of SjAQP. (**A**) *Sj*AQP-expressing oocytes significantly facilitate glycerol movement across oocyte membranes compared with control oocytes (**B**) *Sj*AQP-expressing oocytes significantly facilitate urea movement across cell membranes compared with control oocytes. The *x*-axis is the coefficient of solute permeability, P_s_, with the unit 10^−6^ cm/s. Data are represented as means ± SDs. **p ≤ 0.01 by a Student’s *t*-test.

cRNA of myc-tagged *Sj*AQP was transcribed *in vitro*. The size and quality of the cRNA were confirmed, and then this cRNA was injected into *X. laevis* oocytes to express the *Sj*AQP protein. Osmotic swelling assays showed that the P_f_ in *Sj*AQP-expressing oocytes was 21.9-fold greater than that of the controls. Water permeation through *Sj*AQP was significantly reduced to only 31% of its natural level by incubation with 1.0 mM HgCl_2_, a typical AQP inhibitor, while control oocytes were not significantly affected by HgCl_2_ (Fig. 2A). We also found that certain non-mercurial blockers significantly inhibited the water-permeating activity of *Sj*AQP. After the addition of 3.0 mM tetraethylammonium, 1.0 mM copper, or 1.0 mM zinc ions, *Sj*AQP-expressing oocytes only exhibited 42, 37, or 39%, respectively, of their full activity (Fig. 2B). More interestingly, oocytes expressing *Sj*AQP exhibited glycerol and urea P_s_ values that were 29.8- and 21.2-fold, respectively, greater than those in control oocytes (Fig. 3A,B); thus, *Sj*AQP is a functional aquaglyceroporin subfamily member.

### Expression of *Sj*AQP during the *S. japonicum* life cycle

**Figure 4:**
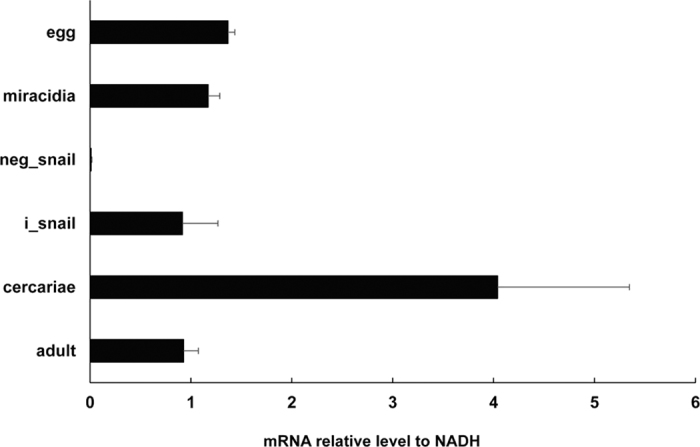
Expression profiles of *Sj*AQP in different stages of the parasite life cycle. Reverse transcription-quantitative polymerase chain reaction revealed the expression of *Sj*AQP in *S. japonicum* developmental-specific stages – eggs, miracidia, cercariae, and adults. cDNA levels were normalized using the NADH housekeeping gene as an internal control. neg_snail, naive snails as the negative control; i_snail, *S. japonicum* infected snails.

Using quantitative RT-PCR with total RNA extracted from *S. japonicum* at different developmental stages, we found that *Sj*AQP mRNA was constitutively present in the egg, miracidia, infected snail, cercaria, and adult stages. Notably, the *Sj*AQP expression level was highest (approximately four times higher than that of the other stages) in the cercaria stage (Fig. 4).

## Discussion

*Schistosoma* spp. undergo tremendous osmoregulatory stresses as they develop from one life stage to another in different environments^29^. The environment of fluke parasites alternates from eggs in the feces of humans and livestock to miracidia and cercariae in fresh water and snails and, finally, to warm-blooded mammalian hosts. It is critical for the parasite to survive dramatic osmolality changes from feces to freshwater snails, and later from snails to hosts. Additionally, blood-sucking parasites take in a huge amount of blood relative to their bodyweights. For example, *S. mansoni* female adults ingested 330,000 erythrocytes per hour, and take in a total fluid equivalent of 4.4 body volumes per day^30^. To survive the extreme osmolality changes between life stages, or to concentrate nutrients and exclude excess water, parasites must develop an effective system to permeate osmolytes and water.

AQP channels are widely expressed in organisms, and they confer unique trans-cellular movements of water and certain small solutes^9^. AQPs in *S. mansoni* and two liver flukes, *O. viverrini* and *C. sinensis*, play important roles in parasite physiology and water equilibrium^31^,^32^,^33^,^34^. Recently, using transcriptomic and proteomic analyses, we found that there is another annotated *Sj*AQP, “MIP” (GenBank accession no. AAW24850.1)^23^. It has 63.0% amino acid sequence identity to our *Sj*AQP; however, there was no functional study of an *Sj*AQP until we reported the results of *Sj*AQP-expressing oocytes in this study. Further investigations are needed to confirm that MIP is a functional AQP.

*Sj*AQP shares slightly higher sequence similarity with orthologs in liver flukes from different genera, *O. viverrini* and *C. sinensis*, compared with *S. mansoni* from the same genus (Fig. 1B). *S. mansoni* mainly resides in South America, the Caribbean, Africa, and the Middle East^35^,^36^, while *O. viverrini* and *C. sinensis* are both common fish-borne liver flukes in Asia, including China^37^; thus, there may be a geographical influence on the molecular evolution of parasite AQPs.

In the oocyte-swelling assay, we observed significantly faster water or solute movements in *Sj*AQP-expressing oocytes, which implied that *Sj*AQP was well expressed and performed its functions. Generation of a specific polyclonal antibody against *Sj*AQP is ongoing, and additional investigations will be performed in the future.

We identified several inhibitors that specifically reduce water permeation through *Sj*AQP (Fig. 2). They have much lower cell toxicities compared with mercurial chloride and, thus, have great potential as new *Sj*AQP antagonists.

Distinct from water-selective orthologs from *O. viverrini*^33^, *Sj*AQP shows permeability to glycerol and urea in addition to water (Fig. 3). During *S. japonicum* development stages, water, glycerol, and urea are all fundamental for parasite physiology. Water accounts for up to 80% of the living organism bodyweight^38^. Glycerol is a building block of membrane synthesis, and it also enters metabolic pathways, such as glycolysis or gluconeogenesis pathways, to provide energy for growth^39^,^40^. The elevated glycerol level in mice following *P. berghei* infection proves the high demand for glycerol during parasite proliferation^41^. Urea is a metabolic waste product that needs to be excreted to maintain normal physiology^21^,^42^. Therefore, as an aquaglyceroporin, *Sj*AQP may be essential for parasite physiology, and it may serve as a novel target to block parasite invasion.

We found an interesting expression pattern of *Sj*AQP, which is constitutive throughout the *S. japonicum* life cycle, with a peak during the cercaria stage (Fig. 4). This differs from *Sm*AQP, which shows peak expression in adults^32^ *S. japonicum* infection occurs when cercariae are released by snails into fresh water, and then cercariae penetrate human skin to achieve invasion^43^. Our finding of peak *Sj*AQP expression in cercariae suggests that *Sj*AQP probably protects parasites from osmolality changes when moving from fresh water (zero osmolality) to hosts and vectors (physiological osmolalities) and vice versa. We would expect that if *Sj*AQP expression was reduced, or if the *Sj*AQP gene was deleted or deactivated, the *S. japonicum* parasite would become significantly sensitive to osmolality changes. A similar trend has been observed in *O. viverrini*, in which suppression of two AQPs disabled water influx into parasites under hypoosmotic conditions^33^.

*S. japonicum* is currently endemic in the low reaches of the Yangtze River in China, and it causes major health problem^8^. Current research focuses on immune genes. However, channel-, transporter-, or metabolism-related genes also contribute to parasite proliferation. For instance, deletion of *Pb*AQP reduces the virulence of a malaria parasite^40^,^44^. A reduction in the expression of a trehalose transporter leads to decreased *P. falciparum* parasite intensity in the vector midgut^45^. The disruption of *P. falciparum* glycerol kinase results in deficient parasite growth^46^. *Hs*AQP8 facilitates hydrogen peroxide transport and mitigates oxidative stress during *Plasmodium* infection in red blood cells^47^. Therefore, *Sj*AQP may be important for parasite invasion, and our study will fill knowledge gaps regarding pathogen-vector interactions during the *S. japonicum* transmission cycle.

## Conclusions

Taken together, we have identified *Sj*AQP as the first aquaglyceroporin from *S. japonicum* that functions *in vitro* and the results suggest its important roles in parasite water and osmolyte equilibria.

## Additional Information

**How to cite this article**: Huang, Y. *et al*. Cloning and *in vitro* characterization of a *Schistosoma japonicum* aquaglyceroporin that functions in osmoregulation. *Sci. Rep.* **6**, 35030; doi: 10.1038/srep35030 (2016).

## Figures and Tables

**Table 1:** Primers used for *Sj*AQP cloning and qPCR.

For Cloning	
*Sj*AQP_F	TCAGAAGCTTATGGGGCGTACTGATACATTTG
*Sj*AQP_R	ACTGAGATCTTTATAATCCCCGAGTTAGTAAG
For qPCR	
*Sj*AQP_qF4	GTTGGTGCACTTGCGATTTATG
*Sj*AQP_qR4	AGATGAGAGGCTCCAGGATTAG
For RT-qPCR quality control	
*Sj*NADH_F	CGAGGACCTAACAGCAGAGG
*Sj*NADH_R	TCCGAACGAACTTTGAATCC
OhTPx_qF3	AGGCTTATGGCGTGTATCTG
OhTPx_qR3	CAGGTCGTTCATGGTGATCT
